# Recent Advances in Flexible Sensors and Their Applications

**DOI:** 10.3390/s22124653

**Published:** 2022-06-20

**Authors:** Bouchaib Zazoum, Khalid Mujasam Batoo, Muhammad Azhar Ali Khan

**Affiliations:** 1 Department of Mechanical Engineering, Prince Mohammad Bin Fahd University, Al Khobar 31952, Saudi Arabia; mkhan6@pmu.edu.sa; 2 King Abdullah Institute for Nanotechnology, King Saud University, Riyadh 11451, Saudi Arabia; kbatoo@ksu.edu.sa

**Keywords:** flexible sensors, robotics, health monitoring

## Abstract

Flexible sensors are low cost, wearable, and lightweight, as well as having a simple structure as per the requirements of engineering applications. Furthermore, for many potential applications, such as human health monitoring, robotics, wearable electronics, and artificial intelligence, flexible sensors require high sensitivity and stretchability. Herein, this paper systematically summarizes the latest progress in the development of flexible sensors. The review briefly presents the state of the art in flexible sensors, including the materials involved, sensing mechanisms, manufacturing methods, and the latest development of flexible sensors in health monitoring and soft robotic applications. Moreover, this paper provides perspectives on the challenges in this field and the prospect of flexible sensors.

## 1. Introduction

**Figure 1:**
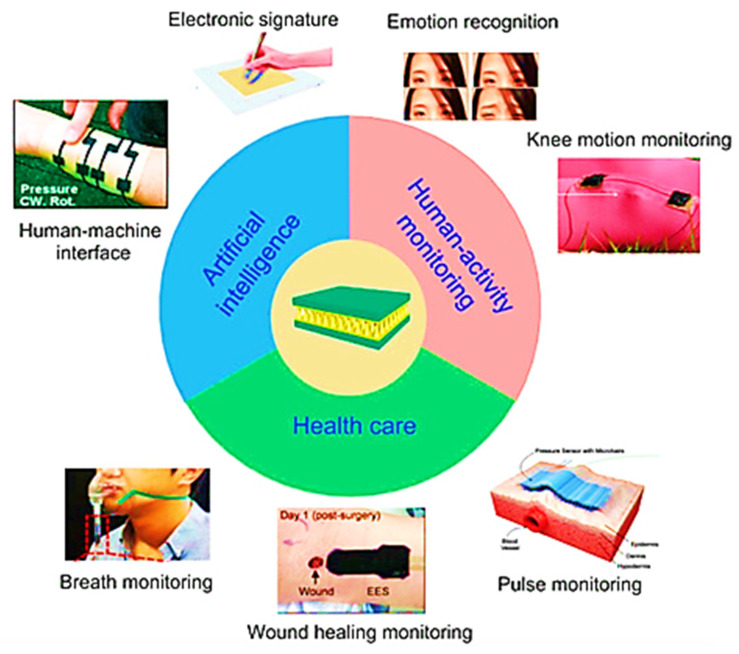
The main parts of a flexible sensor comprise flexible materials and applications [16]. Copyright 2017, Elsevier.

Flexible and wearable electronics devices are usually used for detecting external signals and responding consequently. The rapid development of flexible wearable electronics has led to the expansion of their engineering applications for health monitoring and artificial intelligence. Sensors are considered the important elements of flexible wearable electronics. They are employed for converting physiological signals into electrical signals in health monitoring, including joints bending, blood pressure, and heartbeat. Common electronic sensors are typically made of metals or semiconductors and have limited sensitivity and stretchability; therefore, they are not appropriate for monitoring physiological signals. Compared to the existing conventional electronic sensors, the flexible sensor has shown high biocompatibility and real-time monitoring, as well as further advantages. They are the most extensively utilized in human health monitoring and human-machine interaction [1,2,3,4,5,6,7,8,9,10,11,12,13,14,15] owing to their simple structural design, which converts physical motion, including compression, bending, tension, shear, stress, and strain, into electrical signals. These kinds of sensors can detect human quotidian activities, including joints and muscle movement, respiration, pulses, etc. Therefore, important research efforts have been made to develop a new generation of flexible sensors with promising sensitivity and mechanical flexibility. Figure 1 illustrates some of the great challenges facing flexible sensor development, including materials, fabrication methods, and device applications [16].

The most important factors that evaluate the sensors are sensitivity, flexibility, and lifetime of usage. On the one hand, applications such as joints motion have higher tensile property requirements, whereas pulse monitoring requires high sensitivity, but on the other hand, high flexibility and large sensitivity are two parameters that are often difficult to be achieved simultaneously. Therefore, designing sensors with high mechanical flexibility and high sensitivity at the same time remains a big challenge [17,18], which can restrict their engineering applications in health monitoring and human-machine interactions.

This paper gives an overview of the research evolution of flexible sensors, including the materials employed for flexible sensors, the most commonly used fabrication methods, the sensing mechanism permitting the conversion of physiological activities into electrical signals, and the newest applications of flexible sensors in health monitoring and artificial intelligence.

## 2. Selection of Materials

For the fabrication of flexible sensors, the materials utilized are primarily divided into three main categories: Metallic materials, carbon-based materials and polymers [19]. Each of these three categories will be described below.

### 2.1. Metal Materials

The most common conductor used in force sensors is metal. The metals used in the fabrication of flexible force sensors (FFS) are copper, gold, titanium, nickel, magnesium, silvine, chrome, molybdenum, zinc, etc. [20]. Each of these metals is used in different forms including metallic films, metallic nanomaterials, liquid, metallic oxides, and MXenes.

Metallic films come in a variety of materials such as gold, copper, aluminum, silver, and zinc. Hogas et al. [21] proposed a manufacturing method combining thin film deposition with electrospinning to manufacture a strain sensor. While the sensor did prove its high sensitivity, it had low signal to noise ratio. Thus, the electrode layers of FFS are often made of metal films.

Metal nanosheets, nanowires, and nanoparticles are types of metallic nanomaterials. To address the requirements of high transparency and flexibility, metal nanomaterials with different geometries are used [20]. Metal nanowires are of particular interest owing to their high aspect ratios and thinness. Light transmittance and flexibility are their added advantage. In addition, metal nanowires easily form percolation channels [22]. While there are multiple metallic nanowires, those that demonstrate high conductivity and stretchability are those made of copper, gold, and silver [23,24,25]. However, silver nanowires are the ones used most widely since they offer both good conductivity and antibacterial properties. In contrast, copper nanowires are easily oxidized, and gold nanowires are expensive. Dong et al. [26] used a composite network with a layered structure to develop an elastic piezoresistive sensor. The sensitivity of the sensor was 0.016 kPa^−1^ in a range of 0 to 40 kPa, and the gauge factor (GF) was 1.5. The sensor demonstrated more than 7000 load and unload cycles and a response speed smaller than 54 milliseconds. The sensor was made of silver nanowires that, due to their high aspect ratios, are prone to entanglement with each other under high stress situations; this explains the reason for the sensor’s low sensitivity. Kwon et al. [27] used nanocomposites to make a multi-axis resistance sensor with graphene nanosheets and silver nanoparticles with polyurethane as an elastic substrate. The sensor demonstrated a GF up to 48.2 and a response time just under 72 milliseconds. 

Amorphous metals in liquid form at room temperature are called liquid metals, this makes them suitable for ink printing. Mercury and Eutectic Gallium Indium (EGaIn) are the liquid metals that are used the most [28]. Mercury is toxic and hazardous to health, making it dangerous and thereby unsuitable for making wearable sensors. EGaIn, however, is used in flexible electronics since it is biocompatible [29]. Ali et al. [30] confirmed the linearity of average capacitance change in electrode channels of the sensor that were filled with EGaIn liquid metal. The capacitive sensor demonstrated 0.11% sensitivity with a GF of 0.9975. Liquid metal is on its way toward mass application in the biomedical and wearable electronics industries due to its flexibility, resistivity, and biocompatibility.

Metal oxides have gained enormous interest in the development of flexible sensors due to their tunable band gap, low cost, large specific area, and ease of manufacturing [31,32]. They also offer good biocompatibility, high response speeds, and durability in different working conditions. Lee et al. [1] fabricated a flexible strain sensor with zinc oxide nanowires (ZnO NWs) on a textile substrate. The flexible sensor was found to have a gauge factor of around 0.96 and provided channels with high conductivity and outstanding stability toward mechanical deforming. When the nanowires were allowed to grow freely in reduced graphene oxide RGO for 3 h, the GF rose up to 7.64. This proves that zinc oxide contributes to sensitivity. This paves way for the application of metallic oxides in the textile sector to create sensory systems that sense the bending strain of the human body.

MXenes are a class of inorganic compounds made of carbonitride and metal carbides. They can be obtained by selectively etching a 3D layered compound in strong acidic or basic solutions. MXenes are simply denoted with M_n+1_X_n_, where M represents a transition meal and X can be either nitrogen or carbon. MXenes have recently captured attention for the manufacturing of FFS. Sobolciak et al. [33] fabricated a piezoresistive sensor with electrospinning pads using modified MXenes. The gauge factor of the sensor went up to 4.5. MXenes demonstrate good flexibility, are resistive to oxidation, and show high conductivity. However, increasing the range of the sensors made with MXenes is still under research.

### 2.2. Carbon-Based Materials

These kinds of materials are used in the manufacturing of flexible force sensors primarily due to their outstanding conductivity, lightness, stability, and flexibility. Mostly, these materials include carbon black, carbon nanotubes (CNTs), and graphene and its oxides [19]. These materials are often mixed with polymers, forming composites with high levels of conductivity.

Carbon black is a form of carbon that has an amorphous structure similar to graphite [34]. Carbon black, albeit low in cost, is a conductive nanoparticle. It can be used to increase the strength and electrical conductivity of materials by adding it in the elastic matrix of composites [35]. When silicone elastomer Eco-flex substrate is layered with carbon black, the sensor thus formed showed a high gage factor up to 3.7, stretchability of 500%, and high repeatability (cycle up to 10,000). It was also found to have reduced hysteresis [35]. In one study [36], a porous structure formed using carbon black and NaCl in TPU (Thermoplastic Polyurethane) created a sensor with high sensitivity of 5.5 kPa^−1^ and a wide range of pressures that could be measured with it. For wearable sensors, carbon black is an attractive candidate due to its affordability and high conductivity.

Different allotropes of carbon fall under this category. These are, to simplify, layers of grapheme rolled to form a tubular shape [37]. In 1991, multi-walled carbon nanotubes were reported and in 1993 [38], CNTs’ single walled counterparts were reported [39]. Carbon nanotubes, due to their easy synthesis, affordability, and chemical stability, have captured a lot of interest [40]. Using multi-walled carbon nanotubes, Abshirini et al. [41] synthesized a strain sensor with a GF of 4.3, high stability, and low strain rates. Single walled carbon nanotubes, however, have higher surface area per unit mass, strength and uniformity compared to multi-walled carbon nanotubes. Using dry-transfer manufacturing, Gilshteyn et al. [42] manufactured strain sensors based on single walled carbon nanotubes. The sensor had high levels of transparency, sensitivity, and conductivity. The tunneling resistance between nanotubes can be varied, making piezoresistive composites made with CNTs highly sensitive. Their durability, however, is a shortcoming. Their conductivity decreases over time due to cracks in the tube structure.

Graphene is known for its good conductivity and high surface area for its unit mass [43]. However, it is difficult to be fabricated. Graphene oxide is a derivative of graphene and holds functional groups containing oxygen. It is viscoelastic in water solution and has high viscosity and printability. A resistive strain sensor with a conductive network structure in 3D was fabricated by Wang et al. [44]. The sensor demonstrated high sensitivity, repeatability and response rate. Wang et al. [45] also proposed that controlling the sensor’s microstructure could work as a viable way of adjusting the sensor’s GF, and fabricated a graphene strain sensor with a porous structure. Such a sensor, primarily due to its adjustability, can be used in wearable health monitoring appliances as the microstructure of the sensor could adapt to stress conditions of body parts.

### 2.3. Polymer Materials

Polymers are commonly used as supporting materials for sensors. Common polymers with flexible properties are polydimethylsiloxane, polyimide, polyurethane, polyethylene terephthalate, polyvinylidene fluoride, parylene, polyethylene naphthalate, etc. They are known for their strength, thermal conductivity, ease of compounding with conductive materials, and chemical stability [46]. Resistive sensors and active electrodes are made by filling conductive active materials into an elastic polymer matrix. Rycewicz et al. [47] proved the preparation of boron-doped diamond nanosheets (BDDNS) on Kapton as polymer substrates. The fabricated BDDNS/Kapton sensor was capable to measure up to 0.55% of strain, which shows it may have a high chance to be used in low-strain sensor application.

## 3. General Design and Categories of Flexible Sensors

The material selection and design of devices are vital to sensors. Traditionally, piezoelectricity, piezoresistivity, and capacitance have been used as transduction methods. Tribo-electricity is a relatively novel mechanism to sense minuscule stimuli, and it is under extensive research to broaden its applications.

### 3.1. Piezo-Resistive Sensor

This sensor is used due to its simple design and reading method. It allows force variations to be transduced into easily detectable changes in resistance [48,49,50,51,52]. Changing the path of conductance in an elastic composite and changes in contact resistance are common methods for determining resistance dependence for force-sensitive sensors.

**Figure 2:**
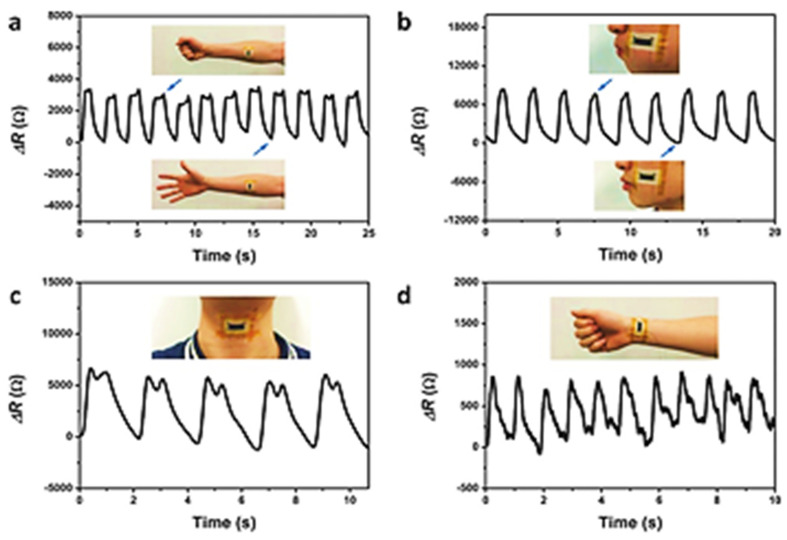
Application of the AuNWs/latex strain sensor in human motion monitoring: (**a**) movement of the forearm muscles; (**b**) movement of the cheeks; (**c**) continuous throat movement while saying “hello”; and (**d**) detecting human wrist pulses [66]. Copyright 2015, John Wiley and Sons.

Many research projects use piezoresistive mechanisms and involve the use of conductive nanomaterials with flexible substrates. Tactile sensors with high sensitivity are made quite effectively with gold nanoparticles. They have been used to make stretchable strain sensors [53,54,55,56,57]. For health monitoring systems, gold nanowires (AuNWs) were integrated with latex rubber to fabricate AuNWs/latex strain sensor [58]. Figure 2 displays the performance of these sensors when employed for human motion detection. These nanowires can range anywhere from two nanometers to several tens of micrometers in length without any significant compromise in their mechanical flexibility. The sensors show stretchability of more than 350% for a GF of around 10%, a response time faster than 22 ms, and a tensile strain from 0.01% to 200%. These sensors can not only be utilized for monitoring limbic movement but also effectively measure pulses. When used in a glove, the sensor can distinguish between hand positions and gestures. Wearable e-skin sensors were also prepared by integrating graphite nano-plates into polyurethane [59]. At a strain variation of 30%, the sensors have a GF of 0.9 and are capable of detecting forces as low as 5 mN both in static and dynamic situations. The sensors were also found to have a linear response against elongation and bending, making them more suitable for healthcare applications. A different e-skin sensor using a few layers of graphene with PVDF substrate showed a GF of 320 at 2% strain which drops down to 67 at 0.2% strain [60]. This sensor can detect small stimuli and tiny deformations due to its high GF. The sensitivity, as well as the sensor’s stretchability, can be increased even further by using conductive porous structures with good mechanical and electrical properties. The pressure sensor made of a 3D hybrid network of multiwalled carbon nanotubes demonstrated good elasticity and high sensitivity along with high durability. The response time of the sensor was 45 milliseconds. This allowed the sensor to measure precise human motions such as coughing and breathing [61]. Other porous materials such as 3D Grapheme are also used for making piezoresistive sensors [62,63,64,65]. A list of gauge factor GF values for several materials can be found in Table 1.

### 3.2. Capacitive Sensor

**Figure 3:**
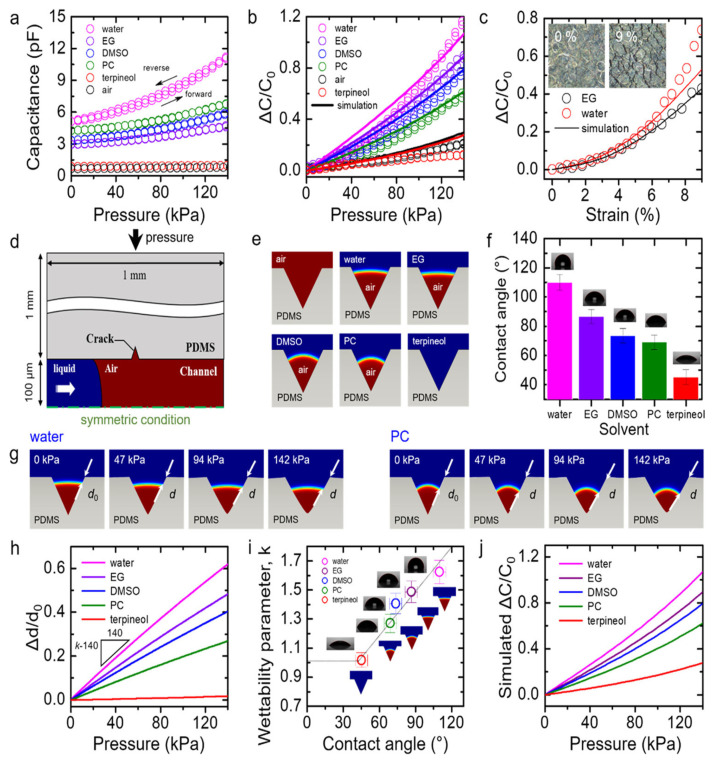
(**a**) An analysis of the capacitance at 0–140 kPa as a function of applied pressure for crack-enhanced microfluidic pressure sensors prepared with five sensing liquids. (**b**) Normalized capacitance plotted as a function of all sensors. The values simulated are represented by solid lines. (**c**) Normalized capacitance of EG or water-based sensors as a function of train, ranging from 0% to 9%. (**d**) The channel and crack simulated in the COMSOL are depicted schematically in this diagram. (**e**) In each liquid example, COMSOL modeling results were achieved under the initial conditions, simulating the interface in the crack after filling the channel. (**f**) Angles of contact between liquids and the PDMS surface. (**g**) COMSOL modeling results produced by COMSOL for the water and PC under various pressure settings. (**h**) Simulated Δd/d_0_ vs. the applied pressure corresponding to each. (**i**) Wettability parameters (k) plotted as function of contact angle. (**j**) Illustration of the simulated normalized capacitance as function of the pressure [83]. Copyright 2017, American Chemical Society.

Capacitance can measure the storage of charge. For parallel plate capacitors, it is a function of the area of the plates, the distance between the plates, and the dielectric constant. When force is applied to a capacitive sensor, the distance or the area of the plates changes, and the capacitance changes accordingly. Employing this principle, wearable sensors can be made using nanomaterials that measure capacitive strain [73,74,75,76,77,78,79,80]. A novel capacitive strain sensor using CNTs demonstrates good durability with a strain range between 1% and 300% [81]. The sensor also shows a linear capacitive relation and high sensitivity. Similarly, a textile-based pressure sensor was fabricated [82]. This sensor was prepared by coating an elastic form of rubber with silver nanoparticles. It showed good stability of more than ten thousand cycles, response times below 10 milliseconds, and sensitivity of 0.21 kPa^−1^. This sensor is quite promising for the textile sector as it can be integrated with clothes and gloves. Another type of this sensor was made in the microfluidic form (Figure 3) [83,84]. The sensor was made of rubber-based microfluid channels placed between layers of silver nanowires. The layer of rubber channels is deformed when stress is applied and microcracks form on its surface. This allows the fluid to penetrate the channel, thereby altering the dielectric properties of the material and thus changing capacitance. The sensor can measure pressure ranging from 0.1 to 140 kPa and is able to detect the movements of muscles. The sensitivity depends on the initial fluid interface and the length between the liquid and the rack wall. Capacitive sensors can also be fabricated into transistors. These transistor-based capacitive sensors are affected by the gap between the layers, which changes rapidly as pressure is applied to the sensor [85]. These sensors are very sensitive, and their signal-to-noise ratios are high as transistors amplify the signals relative to noise. The dielectric properties are read out in the form of AC impedance [86]. The sensors are formed by printing graphene oxide materials using a laser. Electrodes are formed by reducing the written area to conductive materials. The fabricated sensor has a fast response time, recovers quickly, has high sensitivity, and is quite robust.

### 3.3. Piezoelectric Sensor

A power supply is needed for flexible sensors that use piezoresistivity and capacitance. This makes them impractical. The piezoelectric effect generates electrical signals that are used to power the sensor. The force applied to a piezoelectric material (PZT) generates a voltage. These PZT materials are used as pressure or strain sensors as they can convert vibrations or applied pressure into electrical signals [87,88,89,90,91]. Fish gelatin nanofibers are made for flexible sensors using electrospinning technology. This has produced affordable, eco-friendly, flexible and lightweight sensors [92]. Owing to the stability and improved mechano-sensitivity of these nanofibers, the flexible sensors can power their functions and can emulate human sensations with a reasonable lifetime of up to 6 months.

Poly(L-lactic acid) nanofibers can also be used to create wearable sensors with piezoelectric properties. These sensors can study human signals and detect dynamic stimuli [93]. By aligning molecular orientations while electrospinning, the piezoelectric charge coefficient is improved. The resulting sensor produces 22 V/N of force applied to it. This enables the sensor to effectively monitor subtle muscle internal movements. Since the nanofibers have high mechanical strength, they can operate for more than 375,000 cycles. With NiO/SiO_2_/PVDF nanocomposites, sensors with very high sensitivity were produced [94]. These kinds of sensors could detect dynamic and static pressure distributions in the human body. Piezoelectric sensors have been reported to be able to detect glucose levels in bodily fluids [95]. Under deformation, the voltage generated by the sensors was observed to be significantly affected by glucose levels. The experimental validation opens up new research avenues for monitoring body fluids.

### 3.4. Triboelectric Sensor

**Figure 4:**
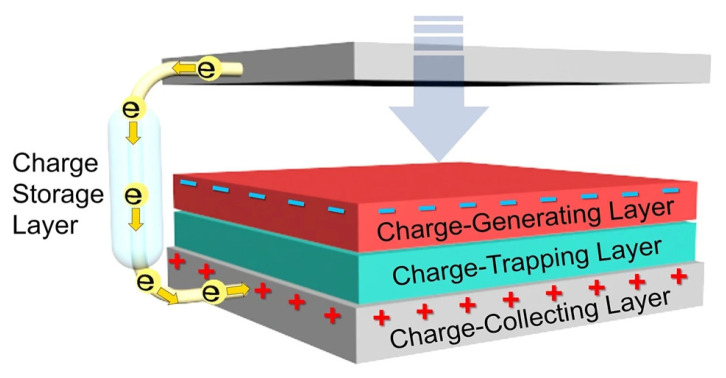
Sketch of main components of a triboelectric nano-generator, including the charge-generating layer, the charge-trapping layer, the charge-collecting layer, as well as the charge-storage layer. Reproduced with permission [109]. Copyright 2020, Springer Nature.

Besides piezoelectricity, triboelectric phenomena have been demonstrated in nano-generators (Figure 4) to create self-powered sensors [96,97,98,99,100,101,102,103,104]. Both electrostatic induction and the triboelectric effect are used in triboelectricity to convert mechanical input into electrical signals. The amount of electricity generated can change with different conditions including time and area, each affected by mechanical interference. The positive charges are induced on a human finger as it touches the sensor surface, while the negative charges stay on the surface of the sensor for a long time as the surface is insulating, and the charges cannot be induced outside easily. When the finger is removed from the surface, the positive charge from the finger is induced onto the electrodes. The induced current flows in and out of the electrodes as the human finger approaches or moves away from the device. This forms the principle behind self-powered pressure sensing. The sensor thus formed has a fast response time of 68 milliseconds and can measure extremely small pressure ratings. Another self-powering sensing array was made of nanotexture enhanced nano-generators that used triboelectricity [105]. The sensor produces stable voltages and maintains its stability of operation in aqueous environments as well. The sensor can generate a voltage of up to 1.613 V in an open circuit, and in a short circuit, it produces a current of 47.31 mA/m^2^ under a pressure value of 612.50 kPa. Another flexible sensor that was inspired by fingertips was made with a triboelectric sliding sensing mechanism [106]. It could sense sliding pressure caused by friction as well as normal stress applied to it. The voltage frequencies generated against different forms of external stimuli were different, i.e., sliding and pressure. By embedding ZnO arrays on a PDMS substrate, similar sensors were made [107]. The resulting sensor could distinguish between different mechanical inputs such as force applied normally, bending moment, and twisting action. It was implemented and validated by application on a human wrist. More research work on self-powering triboelectric sensors can be found in the review paper [108]. A particularly interesting avenue for research is the integration of piezoresistive sensors with triboelectric sensors. The added porosity in the structures increases the sensitivity of the integrated sensor thus formed. This allows for broader applications, such as the detection of roughness.

## 4. Applications of Flexible Sensors

### 4.1. Flexible Sensors as Biomedical Devices

**Figure 5:**
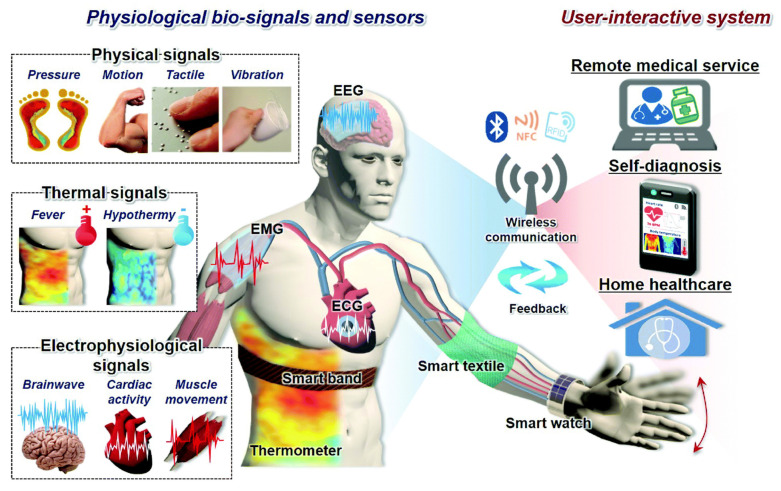
Human body biosignals and the healthcare sensors that measure them. Reproduced with permission [120]. Copyright 2018, Royal Society of Chemistry.

**Figure 6:**
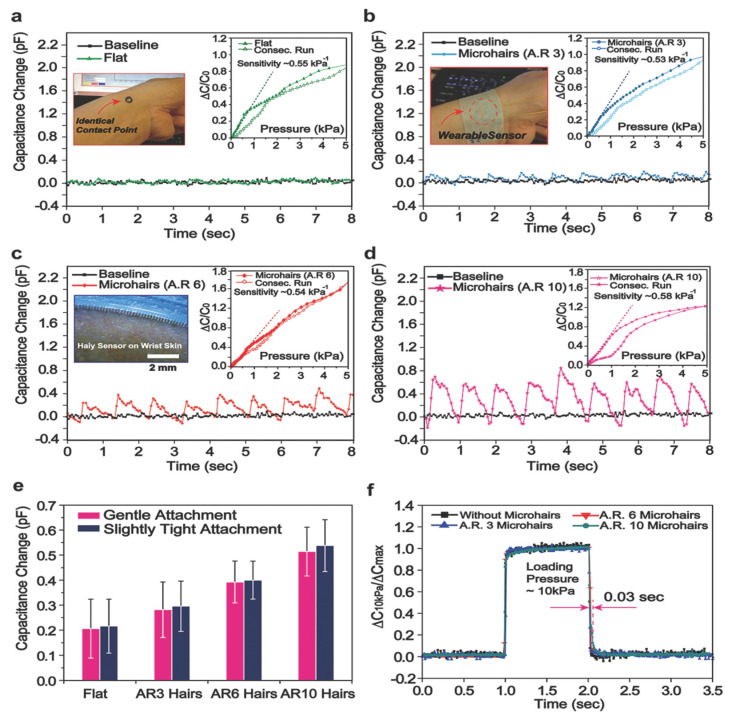
Radial artery pulse waves and characterizations of the capacitive pressure response. (**a**–**d**) The radial artery pulse waves were monitored using four different types of devices with varied geometry. (**e**) Statistical data on the change in capacitance caused by applying varying amounts of shear pressures to different sensors. (**f**) Curves of relaxation and steady state after loading and unloading different types of sensors [121]. Copyright 2014, John Wiley and Sons.

Generally, conventional biomedical equipment is rigid and cannot be fitted closely at the monitoring site, therefore resulting in inaccurate monitoring data and making the patient uncomfortable while wearing a device. Flexible sensors are easily fitted onto human skin due to their great flexibility and stretchability [2,110,111,112,113]. These sensors possess high sensitivity, stretchability, and quick response time, due to which they would be a sustainable and ideal choice for future applications in the biomedical field such as pressure detection, sound variation, vital sign monitoring, and pulse monitoring (Figure 5). Mainly flexible sensors also possess excellent biocompatibility, which could minimize patients’ immune rejection. When two dissimilar surfaces join with each other, they inevitably separate after a certain period [114]. Signal transmission becomes inaccurate after the separation of the sensor from the skin. Adhesives used in traditional sensors can lead to skin infections. As compared to traditional sensors, flexible sensors do not use adhesives and make dry contact through intermolecular Van der Waals forces [115]. For example, to improve the adhesive property, self-adhesive materials and ultrathin electrodes were used to manufacture the flexible sensors. An exceptionally thin parylene-encapsulated Au electrode was created to improve skin adhesion by reducing bending stiffness, resulting in the formation of real-time accurate motion of physiological signals [116]. The addition of a minimal amount of surfactant other than the ultrathin film could also enhance the strength of the adhesion of sensors. There is the use of hydrogel in other methods to manufacture flexible sensors that do not leave any residue after disassembly. Hydrogels possess excellent adhesive and high-durable properties, which make them an excellent conglutinating agent for accurate and precise real-time communication of the physiological signal [117]. Flexible sensors based on hydrogels offer stretchability of up to 2100% and enable detection of large human motions to minute physiological signals. The adhesion performance shows how much the sensor can move with the skin, whereas sensitivity is also a significant characteristic and parameter that depicts the responsiveness of the sensor. Pulse is an essential biological indicator in terms of representing human health. The pulse beat is relatively weak, and it requires the more sensitive detection sensor. Bao et al. [118] made a micro-hair structure-based flexible sensor whose micro-hair structure enables it to adhere and attach better to skin. It also has excellent signal amplification that can enhance the signal-to-noise ratio by more than 12 times. These devices have huge potential in the future in the medical field with evidence that four different types of instruments with definite shapes are used measuring waves in the radial artery, and these devices have shown excellent sensitivity, as shown in Figure 6. The flexible sensors with high sensitivity would play a promising role in weak signal monitoring, and also has future prospect in the medical field. Jer-Chyi Wang et al. fabricated flexible pressure sensor composite film [119]. During intracranial surgery, this miniaturized pressure sensor could rapidly monitor the pressure of a rat’s brain by the implantation of a sensor in the rat skull. The pressure sensor also has huge potential in the utilization of monitoring the sound variation. It could easily identify heavy-metal music and classical music. This pressure sensor could be implanted into an ear with a size of 0.2 cm. Flexible sensors are also integrated with stretchable, soft and curvilinear human tissues with wearing comfort and high monitoring performance. Flexible sensors also have advancements in the biomedicine field such as cost reduction and mass production, and offer improvements in the monitoring of physiological data.

### 4.2. Flexible Sensors as Wearable Device

Flexible sensors could be integrated with stockings, gloves, or skin to monitor and communicate with various signals of the human body [122,123]. Due to development and advancements in living standards, the market size for such wearable devices is constantly growing. The flexibility and tensile characteristics of flexible sensors overcome the demerits of traditional wearable devices. Flexible sensors have the potential to become an integral part of wearable devices due to their biocompatibility, portability, and small volume.

For real-time monitoring of human motion, wearable devices are vital with good stretchability and sensitivity. In reference [124], a graphene-based flexible sensor has been fabricated using a layer of stretchable yarns and PVA. Excellent sensitivity and stretchability (up to 150%) have been displayed by this sensor. It also enables the detection of human motions on a large and small scale, including phonation, joint movement, breathing, and swallowing. When flexible sensors are attached at the elbow, various bending angles can be measured, and when the sensor is adhered at the throat, several phonations can be precisely monitored. Reference [125] used carbon nanotubes to fabricate a flexible sensor. The results showed that the sensor possesses large stretch (more than 900%), fast response, high sensitivity, and long durability. Researchers in reference [126] prepared a flexible nanocomposite sensor by mixing 2D Mxenes with PVA hydrogel matrix, which possessed instant recovery ability, high sensitivity, and good adhesion. The initial length of the 2.5 cm of the nanocomposites can be easily extended to more than 86.0 cm, with 3400% stretching. Roh et al. [127] fabricated a transparent flexible sensor. Various facial responses and expressions such as crying and smiling could be detected and transmitted by sticking the sensor to the face. In a similar way, when people produce different sounds, the throat vibration is distinct in each case. In reference [128], a flexible sensor was prepared. It has the ability to distinguish different phrases of the speaker when adhered to the throat. Additionally, in reference [129], the authors used indium zinc oxide (IZO) as semiconductor nanomembrane for preparing thin and stretchable miniature flexible sensor. Their results showed that the flexible sensor has ability to respond to numerous stimuli, such as temperature, strain, and ultraviolet rays.

### 4.3. Flexible Sensors for Soft Robotics Applications

Traditional robots do not possess sensitivity and flexibility. Due to the arrival of flexible sensors, software robot development has been promoted. Deimel et al. [130] used flexible materials to fabricate a robotic hand that was able to withstand certain impacts and pick up different shapes of objects. Qiguang et al. [131] fabricated a soft tubular actuator which was made of a liquid crystal elastomer (LCE) and also manufactured an untethered soft robot and a multifunctional soft gripper. LCE tubular actuators were made by sandwiching a heating wire between two loosely cross-linked LCEs, rolling them up into a tube, and then exposing them to ultraviolet light. The heating wire is linked with an external voltage source to produce joule heat in the wires, which results in the bending and contracting of the cylindrical brake due to a change in the crystalline state of the surrounding material. A voltage source applied to one or more heating wires can control the bending and contraction of the soft tubular actuator. The battery, microcontroller, and LCE tubular actuator can make a soft robot that would be able to walk on flat surfaces and transport various items on top. In reference [132], the authors developed a simple and affordable fabrication method to create a highly stretchable hydrogel resistive sensor for soft robot multimodal perception. The hydrogel resistive sensor can withstand a tensile strain of up to 1200 percent, which permits large deformations of a pneumatic actuator to be tolerated.

## 5. Conclusions and Perspectives

This review provided the state of the art and developments of flexible sensors, including the materials used, sensing mechanisms, preparation methods, and the current applications of flexible sensors in health monitoring and soft robotics. Regardless of the above-mentioned progress, numerous challenges remain ahead before the massive production and application of flexible sensors. Based on the main concerns outlined, excessive consideration has been given to the enhancement of sensor sensitivity by using new functional materials. On the other hand, the performance parameters for flexible sensors, such as sensitivity, linearity, and stability, govern their definite applications. Therefore, developing flexible sensors with promising performance parameters remains a great challenge. In addition, it is also interesting to understand the sensing mechanisms and investigate new sensing mechanisms. In parallel, future efforts should be made on flexible sensors using computational methods and machine learning approaches, along with polymer nanocomposites chemistry, preparation, and characterization, to understand the process−structure−performance relationship of flexible sensors and accordingly enhance their structural design depending on their applications.

## Figures and Tables

**Table 1:** Gauge factor of different materials.

Selections of Materials	Gauge Factor (GF)	Reference
Metal	1.60 to 2.00	[67]
Poly-Si	43	[68]
Poly-SiC	6	[69]
Ag/PDMS	939	[70]
C diamond	2000 to 3836	[71]
Graphene	150	[72]

## Data Availability

Not applicable.
